# The Neuropathology of Gluten-Related Neurological Disorders: A Systematic Review

**DOI:** 10.3390/nu12030822

**Published:** 2020-03-20

**Authors:** Maxine D Rouvroye, Panagiotis Zis, Anne-Marie Van Dam, Annemieke J.M. Rozemuller, Gerd Bouma, Marios Hadjivassiliou

**Affiliations:** 1 Amsterdam UMC, Vrije Universiteit Amsterdam, Department of Gastroenterology and Hepatology, AG&M research institute, 1081HZ Amsterdam, The Netherlands; g.bouma@amsterdamumc.nl; 2 Amsterdam UMC, Vrije Universiteit Amsterdam, Department of Anatomy and Neurosciences, Amsterdam Neuroscience, 1081HZ Amsterdam, The Netherlands; amw.vandam@amsterdamumc.nl; 3 Medical School, University of Cyprus, 2408 Nicosia, Cyprus; takiszis@gmail.com; 4 Amsterdam UMC, Vrije Universiteit, Department of Pathology, Amsterdam Neuroscience, 1081HZ Amsterdam, The Netherlands; jm.rozemuller@amsterdamumc.nl; 5 Academic Department of Neurosciences, Sheffield Teaching Hospitals NHS Foundation Trust, Sheffield S10 2JF South Yorkshire, UK; m.hadjivassiliou@sheffield.ac.uk

**Keywords:** gluten, coeliac disease, neurological disorders, gliadin, ataxia, neuropathy, myopathy, encephalopathy

## Abstract

Gluten-related neurological disorders (GRND) represent a spectrum of neurological manifestations that are triggered by gluten. In coeliac disease, a T-cell mediated enteropathy is triggered by gluten in genetically predisposed individuals. The underlying pathological mechanism of the neurological dysfunction is not yet clear. The aim of this review is to collate existing neuropathological findings in GRND as a means of aiding the understanding of the pathophysiology. A systematic search of the Pubmed Database yielded 188 articles, of which 32 were included, containing 98 eligible cases with a description of pathological findings in GRND. In gluten ataxia, loss of Purkinje cells, atrophy, gliosis and astrocytosis were apparent, as well as diffuse lymphocytic infiltration and perivascular cuffing with lymphocytes. In patients with large-fiber neuropathy, nerve biopsies revealed axonopathy, loss of myelinated fibers and focal and perivascular infiltration by inflammatory cells. Inflammatory infiltrate was also observed in muscle in myopathy and in cerebrum of patients with encephalopathy and patients with epilepsy. Such changes were not seen in skin biopsies from patients with small fiber neuropathies. The findings from this systematic review suggest an immune mediated pathogenesis for GRND. Future research should focus on the characterization of the inflammatory cell infiltrates and identifying target epitopes.

## 1. Introduction

Coeliac disease (CD) is an autoimmune disorder triggered by the ingestion of gluten in genetically susceptible individuals. Classical CD typically presents to gastroenterologists with a combination of abdominal pain, diarrhea, bloating, anemia and other gastrointestinal symptoms [1]. Over the past decades, a shift in presentation has been observed from these classic symptoms to extraintestinal symptoms in children as well as adults [2,3]. A wide range of extraintestinal manifestations has been attributed to CD, changing the classic perception of a disease limited to the intestine, to a multisystem disorder. CD can therefore manifest with dental problems, consequences of malabsorption, skin and neurological disorders [4]. Some patients can develop refractory CD. This is characterized by persistent villous atrophy despite strict adherence to gluten-free diet. Symptoms are often severe with diarrhea, weight loss and severe malabsorption [5].

Cerebellar ataxia and peripheral neuropathy are the most common neurological manifestations [5,6]. These can also occur in the absence of enteropathy and are sometimes referred to as non-coeliac gluten sensitivity, or simply gluten sensitivity [7,8]. Not much is known about the pathogenesis of such neurological manifestations. However, both humoral and cell-mediated immune mechanisms have been proposed. The aim of this systematic review is to analyze the published neuropathology of confirmed cases of gluten-related neurological dysfunction in an attempt to aid our understanding of the pathogenesis.

## 2. Materials and Methods 

### 2.1. Literature and Search Strategy

A systematic search was conducted on May 1 2019, using the Pubmed database. The following terms were applied, searching title and abstract (TiAb): celiac OR coeliac AND neuropathy OR polyneuropathy OR ganglionopathy OR neuronopathy OR ataxia OR migraine OR headache OR myopathy OR myositis OR epilepsy OR “movement disorders” OR encephalitis OR encephalopathy AND pathology OR pathological OR neuropathology OR neuropathological or post-mortem. Complementary to this electronical search, we scanned the reference lists of all included articles to increase the yield of eligible papers. 

Inclusion and exclusion criteria 

Papers were considered for inclusion if they met the following criteria:

1. Original clinical studies
2. Human subject study
3. Pathological studies in patients with coeliac disease or gluten sensitivity, suffering from neurological illness

Papers were excluded in case they met the following exclusion criteria:

1. If there was no histopathological description of neuronal/brain tissue or muscle
2. No evidence of coeliac disease or gluten sensitivity
3. Reviews, book chapters, editorials
4. Duplicate articles

All abstracts were screened for eligibility. Full articles were read if the abstract was not sufficient in scoring all inclusion and exclusion criteria. In cases where inclusion criteria were questioned, the paper was discussed with at least two authors (PZ & MH) to reach consensus. Careful consideration of the inclusion and exclusion criteria regarding the article in question, led to unanimous decisions of inclusion or exclusion by MR, PZ and MH. 

### 2.2. Ethical Considerations

No ethical approval was needed as this was a systematic review.

## 3. Results

### 3.1. Study Characteristics 

**Figure 1:**
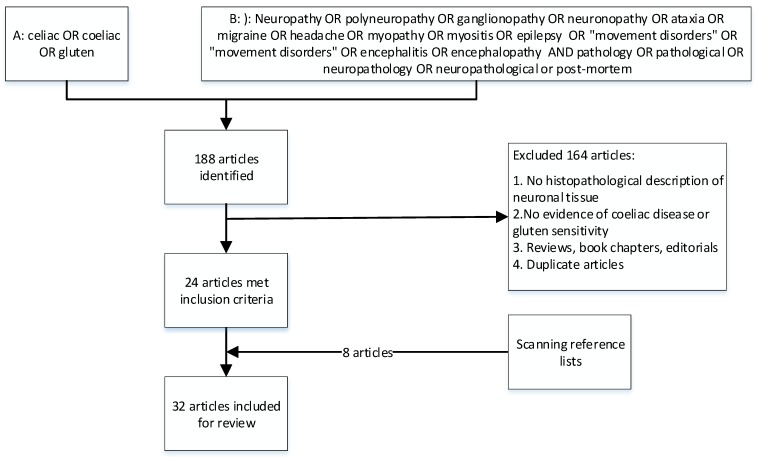
Prisma chart illustrating the literature inclusion/exclusion flow.

The search strategy identified 188 articles. After detailed assessment, 24 papers met the inclusion criteria. Eight additional articles were identified by scanning reference lists of the included papers. In total, 32 articles were included in this review (Figure 1). 

All studies described in the articles were categorized by neurological disorder. Study characteristics and main neuropathological findings are summarized in Table 1. 

### 3.2. Ataxia and Gluten

**Figure 2:**
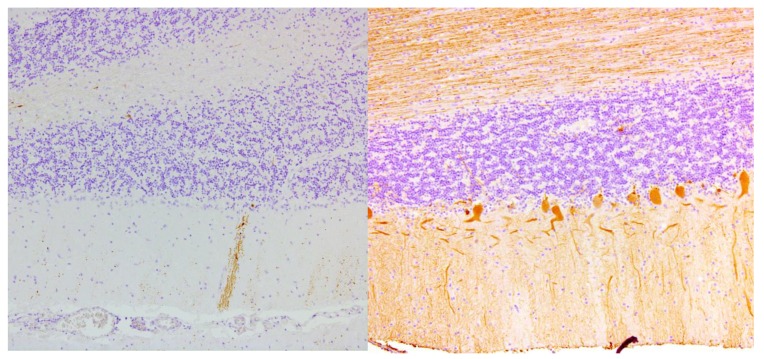
Complete loss of Purkinje cells in the cerebellar cortex of a gluten ataxia patient (left). Normal cerebellum from a control patient on the right shows the single layer of Purkinje cells (calbindin and hematoxylin staining, magnification 100×).

Ataxia refers to loss of coordination, clumsiness and gait instability, and has multiple possible underlying etiologies. Ataxia has been described concomitant with CD and GS [9]. Cooke and Smith where the first to report a case series of 16 patients with established coeliac disease and concomitant ataxia and/or neuropathy. Post-mortem findings were reported in nine cases. Eight patients were male. The diagnosis of CD, based on jejunal biopsy, preceded neurological symptoms by more than two years in seven cases. Five patients had classical symptoms (e.g., weight loss and diarrhea) at the time of neurological presentation. All patients presented with steatorrhea, two patients with anemia, two were diagnosed with osteomalacia and one had a vitamin B12 deficiency. Primary neurological complaints were progressive gait instability, weakness, numbness and pain of the limbs. Adherence to a gluten-free diet did not seem to influence the neurological symptoms. The diagnosis of CD and initiation of a gluten-free diet preceded the diagnosis of ataxia by decades in seven patients. Moreover, in patients with simultaneous diagnosis of ataxia and CD, neurological symptoms slowly progressed despite the initiation of a gluten-free diet. However, no data on strictness of GFD or serology were available in this report. The median age at time of death was 51 years. In six out of nine cases, cerebellar neuronal atrophy and loss of Purkinje cells was demonstrated, in five cases accompanied by gliosis, especially in the dentate nucleus [8]. The loss of Purkinje cells in a patient with gluten ataxia is illustrated in Figure 2. Pathological findings of the cerebrum were neuronal atrophy, gliosis and chromatolysis of the cortex. The inferior olives, the thalamus and hypothalamus showed gliosis and atrophy as well. Demyelination of the posterior columns and anterolateral columns of the spinal cord was demonstrated in eight cases. The spinocerebellar tracts were heavily affected in three patients. Perivascular lymphocytic cuffing, chromatolysis and sudanophil lipophages were demonstrated in various areas of the central nervous system. 

In another case report a 27-year-old female with a slowly progressive ataxia, dysarthria and myoclonic jerks of the limbs was described [10]. Further investigation revealed elevated anti-gliadin antibodies and subtotal villous atrophy with inflammatory infiltrate in the lamina propria. 

Bhatia and colleagues reported four cases of progressive myoclonic ataxia developing after the diagnosis of CD [11]. Autopsy was performed in one patient (male, 68 years old at time of death). He was diagnosed with dermatitis herpetiformis in his twenties and had been successfully treated with medication. Neurological complaints were first apparent at the age of 41 years, with progressive involuntary jerking of the limbs, gait ataxia and cognitive dysfunction. Despite strict adherence to a gluten-free diet, villous atrophy persisted and neurological symptoms progressed. No serological evidence of strict gluten-free diet was available. At 68 years of age, the patient committed suicide. Autopsy revealed cerebellar atrophy and histological examination demonstrated loss of Purkinje cells, Bergmann gliosis, astrocytosis mainly in the granular layer and dentate nucleus. Neuronal loss and astrocytosis was seen in the inferior olives as well. 

Finelli [12] and Kinney [13] reported similar cases with CD and myoclonic ataxia. Both patients were male and had a progressive cerebellar syndrome starting in their fifth and sixth decades, respectively. Histological examination demonstrated severe loss of Purkinje cells and granule cells, gliosis of the dentate nucleus and inferior peduncles. In the spinal cord, demyelination of the anterior and lateral corticospinal tracts, as well as of the posterior columns and lateral corticospinal tracts was also observed. In a series of patients with CD and ataxia published by Hadjivassiliou and colleagues, two autopsies were reported [14]. The findings in one of the patients were dominated by Purkinje cell loss, astrocytic gliosis, vacuolation of neutrophils, diffuse infiltration of lymphocytes and perivascular cuffing by T-lymphocytes in white matter of the cerebellum. Infiltration of lymphocytes was also demonstrated in the posterior columns of the spinal cord. No abnormalities were found in the cerebellum nor in the cerebrum of the second patient. However, there was evidence of spinal cord degeneration of the posterior columns suggesting that the etiology of the ataxia was due to posterior column sensory loss (sensory ataxia). 

Mittelbronn et al. were the first to attempt to characterize the nature of the lymphocytes infiltrating the CNS of a patient with gluten ataxia [15]. In addition to the reported loss of Purkinje cells, granular cells and loss of neurons with accumulation of corpora amylacea in the inferior olives, widespread diffuse infiltration and perivascular cuffing of lymphocytes was reported. Inflammation was characterized by the presence of CD8+ and granzyme B+ lymphocytes and by microglial cell activation. No CD20+ or CD138+ cells were observed. However, in a case described by Nanri with cerebellar atrophy, Purkinje cell loss and mild Bergmann gliosis, no lymphocytic infiltration was observed (CD3-, CD4-, CD8-, CD20-, CD68-, CD79A-) [16]. In a case of a 32-year-old female that died from a rapidly progressive ataxia and neuropathy, CD was diagnosed at the time of neurological presentation [17]. During autopsy capillary changes were found in the white matter, hippocampus and olives marked by vascular and perivascular inflammatory cell infiltrates (CD68+ cells and a smaller CD45Ro+ cell population). Purkinje cell loss and Bergmann gliosis were marked in the cerebellum and loss of neurons was observed in the inferior olives. Further examination of the spinal roots revealed inflammatory cell infiltrates of lymphocytes and macrophages.

### 3.3. Large Fibre Neuropathy and Gluten Sensitivity 

Peripheral neuropathy results from damage of the peripheral nervous system. The underlying pathological mechanism can be driven by hereditary factors, drug-induced damage, infection, inflammation and metabolic conditions such as diabetes and vitamin deficiencies. The cause of neuropathy in the context of CD and GS has frequently been attributed to malabsorption and inflammation. 

Cooke and Smith reported 11 cases with CD and peripheral neuropathy [9]. All but two patients were diagnosed with CD years prior to their neurological diagnosis. The median age at neurological presentation was 49 years and 64% were male. The patients complained of numbness, tingling and pain of the lower extremities and gait instability. Three patients lost their ability to write. In the workup of their neuropathy, low serum vitamin B12 was found in three cases. Osteomalacia was demonstrated in five cases, and three patients tested positive for toxoplasmosis. Muscle biopsies (flexor digitorum sublimis, palmaris longus and flexor carpi radialis) were performed. Tissue containing terminal nerve bundles were then further processed and studied using electron microscopy [18]. Common findings were axonal swelling, loss of myelinated fibers, focal proliferation of sarcolemmal nuclei and collateral re-innervation. 

Chin et al. described three patients that presented with distal paresthesia and dysesthesia [19]. Diagnosis of CD was previously established in two cases and simultaneously diagnosed with the neuropathy in the third. Only one patient adhered to a strict gluten-free diet at the time of presentation. All patients underwent sural nerve biopsies, demonstrating mild to moderately severe chronic axonopathy with loss of myelinated fibers. 

Squintani reported a case of polyneuropathy in a 49-year-old male that presented with painful paresthesia and progressive generalized muscle atrophy, leading to gait instability [20]. Blood tests showed hepatitis B infection. Consequently, his neurological complaints were attributed to a secondary vasculitis. After two years of disease progression, additional tests revealed high antibody levels of anti-gliadin and anti-transglutaminase 2 (TG2). The patient was also positive for endomysium antibodies. The diagnosis of CD was confirmed with a duodenal biopsy. A sural nerve biopsy demonstrated loss of myelinated axons and axonal degeneration. Mild perivascular mononuclear cell infiltration of epineural blood vessels but without fibrinoid necrosis was seen. Nerve fascicles showed degeneration and the perineurium was abnormally thick suggestive of ischemic damage. A year after commencing a gluten-free diet, and six months after steroid treatment, the symptoms improved dramatically. 

Simonati reported the findings of a nerve biopsy performed in a three-year-old child diagnosed with CD that presented with a progressive polyneuropathy [21]. Blood tests showed no evidence of malabsorption resulting in vitamin deficiencies or metabolic disorders. A sural nerve biopsy was performed, demonstrating chronic axonal neuropathy, significant loss of myelinated fibers and Schwann cell nuclei. The density of unmyelinated fibers was low, and no inflammatory cells were identified.

Hadjivassiliou et al. reported on 215 patients with axonal neuropathy [17]. After thorough examination, 140 patients were left with the diagnosis of chronic idiopathic axonal polyneuropathy, 47 (34%) of these tested positive for IgA or IgG anti-gliadin antibodies. Two patients had a sural nerve biopsy. In the first patient a focal inflammatory cell infiltrate in the epineurium was observed, as well as perivascular cuffing of lymphocytes and patchy loss of myelinated fibers and occasional degeneration. In the second patient, no evidence of cellular inflammation was found.

### 3.4. Small-Fibre Neuropathy and Gluten Sensitivity

Souayah reported two cases of confirmed small-fiber neuropathy in patients with CD [22]. The first patient was diagnosed two years following the initial neurological complaints of progressive numbness, tingling and electric-like pains. Skin biopsies of the calf and the thigh were performed. Histopathological examination of the skin biopsies revealed a low to normal epidermal nerve fiber density. Mild qualitative changes were seen in the thigh. Sparse nerve fibers, axonal swelling, increased branching, uneven distribution of epidermal fibers in calf and thigh resembling small-fiber neuropathy were seen. Biopsy results of the second patient showed similar findings. This patient had persistent diarrhea despite adherence to a gluten-free diet for over 17 years, suggestive of the possibility of refractory coeliac disease (RCD).

De Sousa and colleagues characterized 62 patients with sensory neuropathy [23]. In 50% of the cases the small-fiber neuropathy was considered of unknown cause. Eleven of these patients had high CD-related antibody titers. Six patients had biopsy-proven CD. Two of them had morphological changes on skin biopsy. Four also had a reduced epidermal nerve density below the 5th percentile of normality.

Brannagan et al. reported six women and two men with biopsy-proven CD and small-fiber neuropathy [24]. In seven cases neurological symptoms preceded the diagnosis of CD. The epidermal nerve density was measured in number of fibers per millimeter. Distal leg and thigh skin biopsies showed that the density was lower in the distal leg biopsies in five out of eight cases.

**Figure 3:**
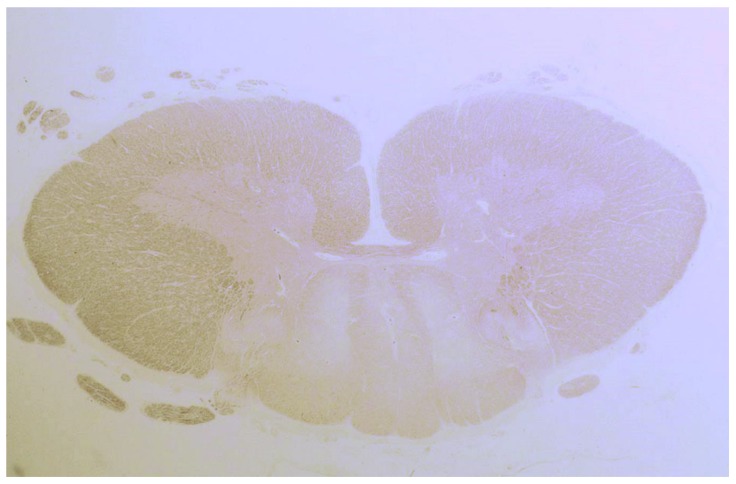
Degeneration (loss of tissue and a pale appearance) of the posterior column of the spinal cord in a patient with gluten ataxia and sensory ganglionopathy.

In another study by Hadjivassiliou et al., seven patients with neuropathy and sensory ganglionopathy were reported [25]. Autopsy was performed on three patients, of which one was previously reported. The other two cases were characterized by spinal cord abnormalities with degeneration of the dorsal columns (Figure 3), and a subtotal loss of myelin, axonal loss and lymphocytic infiltration.

### 3.5. Myopathy and Gluten Sensitivity

Myopathy refers to an abnormality in structure and metabolism of skeletal muscle cells, leading to weakness and often wasting. A CD prevalence of 4.5% in a cohort of patients with idiopathic inflammatory myopathy was reported in one study [26].

Hadjivassiliou et al. described two cases (a 49-year-old female and a 64-year-old male) presenting with progressive pain, weakness, unsteady gait and diarrhea [27]. Muscle biopsies demonstrated an inflammatory myopathy with basophilic rimmed vacuoles suggestive of inclusion body myositis.

Williams et al. reported a similar case of a 51-year-old woman with concurrent CD and idiopathic thrombocytopenic purpura [28]. Muscle biopsy findings were consistent with inclusion body myositis. Henriksson and colleagues reported five patients with polymyositis and adult CD [29]. Muscle biopsies revealed inflammatory cell infiltrate (mostly lymphocytes), muscle fiber necrosis and myophagia.

One patient described by Kleopa et al. had concomitant vitamin E deficiency and an elevated alkaline phosphate [30]. Muscle biopsy revealed inflammatory cell infiltrates and rimmed vacuoles. A sural nerve biopsy showed chronic active axonopathy, loss of myelinated fibers and signs of regeneration and necrotic fibers. All symptoms ameliorated with the initiation of a gluten-free diet. A repeat muscle biopsy only showed residual, mild myopathic features.

A muscle biopsy in a case of CD and myositis described by Alawneh et al. revealed muscle necrosis with neutrophilic infiltration with secondary leukocytoclastic vasculitis consistent with neutrophilic myositis [31].

**Figure 4:**
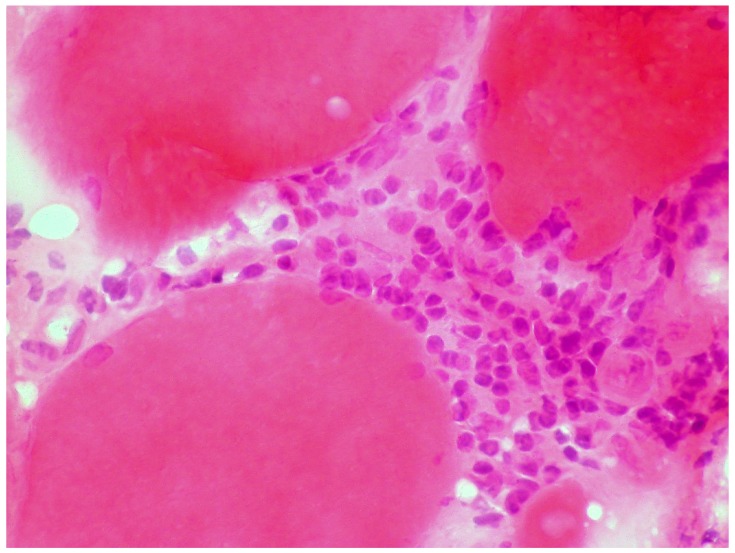
Skeletal muscle biopsy obtained from a patient with gluten myopathy showing myocytes surrounded by lymphocytic infiltration suggestive of an inflammatory process (H&E stain, magnification 400×).

A large case series of thirteen patients with gluten-related myopathy was published in 2007 [32]. The main pathological findings on muscle biopsy included internalization of nuclei, basophilic rimmed vacuoles and fiber splitting, endomysial chronic inflammatory cell infiltrate (CD3+ cells) (Figure 4) and signs of fibrosis in seven cases.

### 3.6. Encephalopathy and Gluten Sensitivity

Encephalopathy is a clinical term implying global brain dysfunction. Patients with encephalopathy can have a spectrum of symptoms ranging from headaches, confusion, disorientation, cognitive deficits, slow mentation and, in extreme cases, altered level of consciousness.

Brücke described a case of a 45-year-old male with progressive loss of energy, arrhythmic myoclonic movements of the arms and tongue, dysarthria, facial palsy and concurrent diagnosis of CD [33]. The patient died a year later from a severe pulmonary embolism. Post-mortem examination revealed brain edema and periventricular lesions with inflammatory necrosis and myelin loss. The inferior olives were hypertrophic and there was gliosis in the vermis with lymphocyte infiltration in the pons and mesencephalon.

Keller and Dimberg presented 2 cases of refractory CD and encephalopathy [34,35]. Refractory CD is defined by persisting malabsorption and villous atrophy despite strict adherence to a gluten-free diet [36]. The neurological symptoms were accompanied by gastrointestinal symptoms characterized by severe diarrhea and weight loss, both patients died within four months after the onset of neurological complaints. Neuropathological findings included loss of Purkinje cells, neuronal loss of the dentate nucleus and perivascular cuffing of lymphocytes. The pons, midbrain, thalamus and basal ganglia showed arterial changes and mild degeneration of the pyramidal tract and posterior columns with loss of myelinated nerve fibers. In the case described by Dimberg neuropathological examination also revealed widespread perivascular lymphocytosis in the cortex hippocampus and temporal gyrus [34,35].

Hu and colleagues reported on thirteen patients with amnesia, personality change, confusion, disorientation, ataxia and seizures who also had CD [37]. Two patients underwent frontal lobe biopsy and three patients underwent post-mortem examinations. These revealed non-specific gliosis and astrocytosis. In a 57-year-old male ubiquitin-positive inclusions were found.

**Figure 5:**
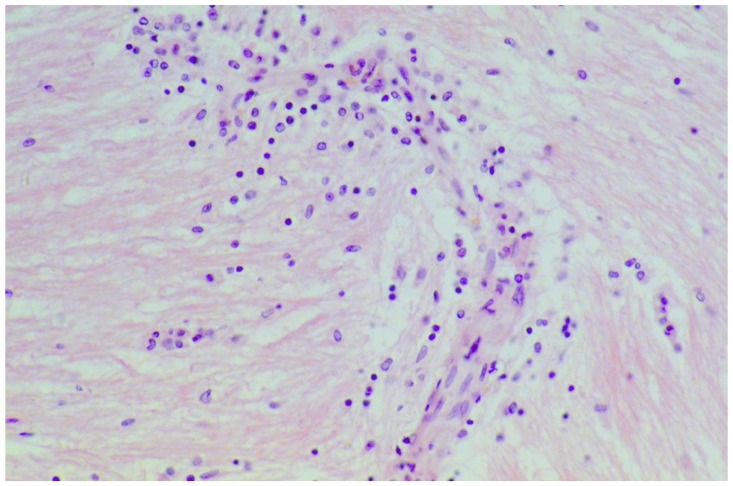
Postmortem cerebral white matter tissue from a patient with gluten encephalopathy showing infiltration with inflammatory lymphocytes and perivascular cuffing (H&E staining, magnification 100×).

La Mantia et al. reported a case of a female patient with intermittent headaches that progressed into chronic headache at the age of 29 years [38]. She was hospitalized five years later with papilledema, peri-papillary hemorrhages and brain swelling. A lobectomy was performed because of a severe intracranial hypertension. Examination of the resected tissue revealed calcifications, increased vascularity, mild neuronal loss, reactive gliosis and demyelination and edematous changes. Coeliac disease was diagnosed at the same time. After the initiation of a gluten-free diet she improved dramatically and was able to resume her job as a teacher. Figure 5 illustrates the infiltration of lymphocytes in the cerebral tissue of a patient that died of gluten encephalopathy.

### 3.7. Epilepsy and Gluten Sensitivity

A wide range of diseases can manifest with seizures [39]. Coeliac disease has been linked to epilepsy. CD is two times more prevalent in epilepsy patients compared to the general population and epilepsy is 1.8 times more prevalent in CD [40]. In some pediatric cases, occipital calcifications can be identified on brain imaging. This combination is termed coeliac disease, epilepsy and cerebral calcifications (CEC) [41].

Two papers have provided neuropathological findings in this group of patients. Bye et al. describe a female patient with CEC and folate deficiency [42]. Her first seizures started at the age of four and worsened in her puberty. Diagnostic evaluation revealed persistent iron and folate deficiency. Subsequently CD was diagnosed, and she started a strict gluten-free diet. An extensive resection of the right lateral occipital cortex was performed. Macroscopically pial angiomatosis was observed, consisting of groups of small veins entrapped by collagen. Severe sclerosis of veins and lymphocytic perivascular cuffing was observed in the cortical neuropil. There was atrophy of the white matter and influx of macrophages, with reactive gliosis. A similar case was reported on a girl with macrocephaly, epilepsy and autistic traits [43]. The girl started having epileptic seizures with episodes of apnea at the age of five months. The seizures gradually developed into generalized complex seizures. At the age of two years CD was diagnosed. She died three years later, probably following an epileptic attack. Autopsy revealed no specific abnormalities apart from megalencephaly.

## 4. Discussion

This systematic review examined the neuropathological findings in gluten-related neurological disorders. Neuropathological findings in the context of gluten ataxia showed loss of Purkinje cells, cerebellar atrophy and gliosis, especially in the granular layer with also atrophy of the dentate nucleus. However, findings were not limited to the cerebellum, but involved other parts of the central nervous system that are closely linked to the cerebellum such as the pons, inferior olives and thalamus.

While vitamin B1, B3, B6, B12, E deficiencies are well known causes of neuropathy and other neurological disorders and can be a consequence of malabsorption due to untreated CD, they are unlikely to be responsible for the pathology described in this review. Furthermore, in almost all cases discussed in this review other possible causes for the neurological deficit, like genetic causes had been ruled out. This review suggests that the pathophysiology of neurological damage in the context of gluten sensitivity has an immune mediated basis.

Whilst there is a female predominance in CD (2.4 F:1.M) and other auto-immune diseases [44], the majority of gluten-related neurological disorders affected men (57%). In the ataxia group this percentage was even higher (76%). The median age at onset of neurological complaints was 50.3 years (excluding the two epilepsy cases with onset at age 6 months and 5 years). The median age at time of CD diagnosis was 44.9 years. There is an increased risk of autoimmunity in individuals diagnosed with CD later on in life [45]. Whether this increased risk can be attributed merely to age and years of gluten exposure is still debated [46,47].

More importantly, in most studies, adherence to a strict gluten-free diet was not monitored. It is therefore unknown whether patients were still exposed to gluten at the time of the development of their neurological dysfunction. [48,49,50,51,52,53].

A consistent finding in three of the ataxia studies was the presence of diffuse infiltrates and perivascular cuffing with lymphocytes in the cerebellar tissue [9,14,15,18]. Lymphocytic infiltration was also demonstrated in several nerve biopsies of patients with neuropathy [17,22,25], muscle biopsies of myopathy patients [26,27,30,32] and in brain tissue of patients with encephalopathy [33,35] and epilepsy [42]. Characterization of these cell infiltrates was only performed by Mittelbronn et al. [15] who described a cytotoxic T-cell population (CD8+ and granzyme B+), and by Souayah (CD68+/CD45ro+ cell populations) [22]. The origin and target epitope of these lymphocytes remain unclear. However, these findings strongly support the notion that the pathology is immune mediated and not related to vitamin or trace elements deficiencies.

It is as yet unknown if and how such cells travel from the intestine to the brain. Crossing of the blood-brain barrier may occur after a compromise due to local inflammation [54]. Nanri et al. [16] hypothesized a humoral response in which anti-gliadin antibodies also recognize epitopes on Purkinje cells. Indeed Hadjivassiliou et al. have demonstrated that anti-gliadin antibodies cross-react with Purkinje cells in vitro. Previously published work has also shown that sera of gluten ataxia patients strongly stain Purkinje cells in cerebellar rat tissue, even after adsorption with crude gliadin [55]. In another study the sera of twenty CD patients and twenty healthy controls were applied on rat brain sections. Sixteen CD patient sera showed immune-reactivity for IgA or IgG on Purkinje cells, deep cerebellar nuclei and brainstem neurons whereas only four sera from healthy controls showed immune-reactivity on the rat brain sections. An additional adsorption experiment with recombinant Transglutaminase (TG2) indicated that anti-TG2 antibodies substantially contribute to neuronal epitope recognition. Of interest is that injection of these antibodies into the lateral ventricle of mice resulted in motor dysfunction. The IgA component from the CD patient sera also cross-reacted with Transglutaminase 3 and 6 [56].

Transglutaminase 6 (TG6), a member of the Transglutaminase family of protein-crosslinking enzymes and is closely linked to TG2 (the autoantigen in CD) and Transglutaminase 3 (TG3, the autoantigen in dermatitis herpetiformis). Transglutaminase 6 has been proposed as the autoantigen in gluten-related neurological disorders [57,58]. IgA deposits against TG6 have been observed in vessels from cerebellar tissue of a patient with gluten ataxia [49]. Moreover, antibodies against TG6 have been detected in the sera of patients with gluten ataxia (73%) and gluten neuropathy (50%), regardless of enteropathy [59,60]. These studies indicate that TG6 antibodies might be helpful in the diagnostic workup of GRND.

The current gold standard for CD diagnosis is based on Transglutaminase 2 (TG2) antibodies (with or without additional testing for endomysium antibodies) followed by histological examination of duodenal biopsies (presence of villous atrophy, crypt hyperplasia and increased intraepithelial lymphocytes). However, even if clinicians consider GRND in a patient with idiopathic neurological complaints, the diagnostic yield using these antibodies is low. This is because the majority of patients with GRND are seronegative for TG2 because they do not have enteropathy [48,49]. Therefore a TG2 antibody test is not sufficient to diagnose GRND [50]. Recent data suggest that a coeliac “lymphogram”, defined as an increase in CD3+ T-cell receptor gamma delta+ (TCRγδ+) intraepithelial lymphocytes (IEL) plus a concomitant decrease in CD3− cells in a mucosal duodenal biopsy, was associated with a sensitivity of 87% (CI, 73.7–95%) and specificity of 96.7% (82.7–99.9%) for CD [52,53]. Therefore it might be worthwhile to assess the intraepithelial lymphogram of duodenal biopsies in (suspected) GRND patients that test negative for TG2 but are positive for TG6 and gliadin antibodies.

Addolorato and colleagues observed a state of hypo-perfusion in multiple cerebral brain regions in untreated CD patients compared to gender and age-matched CD patients on a gluten-free diet and healthy control subjects [61]. In another study, significantly more perfusion abnormalities in the frontal cortex were found in CD patients, regardless of dietary regimen, compared to gender- and age-matched controls [62]. It is unclear if these findings have a bearing on the cognitive deficits observed in patients with gluten encephalopathy.

Three patients, described by Keller, Dimberg (gluten encephalopathy) and Souayah (small-fiber neuropathy), suffering from RCD [22,34,35] had evidence of aberrant intraepithelial lymphocytes that disseminated into mesenteric lymph nodes, blood, bone marrow and other epithelial tissue like skin or lung [63]. It is possible that these aberrant T-cells can also enter the central nervous system. This may explain the observation that ataxia with cortical myoclonus can be a phenotype of RCD that is often extremely difficult to treat both from the gut and the brain perspective. Whatever seems to be driving the gut inflammation seems to also be driving the brain inflammation generating the disabling cortical myoclonus and the ataxia.

Future studies should focus on the characterization of lymphocytes found in brain tissue or CSF and clonality studies may be able to clarify if they originated from the gut. In addition investigating the role of TG6 as a target autoantigen in neurological manifestations may shed light to the pathophysiology of GRND.

## 5. Conclusions

The neuropathological findings in gluten-related neurological disorders are widespread and not limited the cerebellum.

Information on the nature of the lymphocytic infiltration is lacking.

The current evidence is suggestive of both humoral and cell-mediated immunological responses.

More research is needed to further investigate the underlying neuropathological mechanism by characterization of the inflammatory cell infiltrate and identification of target epitopes.

## Figures and Tables

**Table 1:** Study characteristics and main neuropathological findings.

Neurological Disorder Papers *n* First Author/Year/Country	Patients *n*	Tissue	Main Neuropathological Findings
Ataxia 9	18		
Bhatia 1995, UK	1	Brain Spinal cord	Loss of Purkinje cells and cells in inferior olives; Cerebellar gliosis; Astrocytosis of the granular layer, dentate nucleus & inferior olives
Cooke 1966, UK (I) *	9	Brain Spinal cord	Loss of Purkinje cells; atrophy and gliosis of the dentate nucleus, cerebrum, inferior olives, thalamus and hypothalamus; demyelination of post. & ant.-lat. columns; focal perivascular lymphocytic cuffing, chromatolysis and sudanophil lipophages throughout the CNS.
Finelli 1980, USA	1	Brain Spinal cord	Loss of Purkinje and granular layer cells; Neuronal loss & gliosis basal ganglia, inferior olives, substantia nigra; Demyelination ant. & lat. corticospinal tracts
Hadjivassiliou 1998, UK	2	Brain Spinal cord	Loss of Purkinje cells; Cerebellar atrophy & astrocytic gliosis & vacuolation of neutrophils; Diffuse infiltration of lymphocytes & perivascular cuffing of T-lymphocytes in the cerebellum and the post. columns
Hadjivassiliou 2006, UK **	1	Brain	Capillary changes in the white matter, hippocampus and olives marked by vascular and perivascular inflammatory cell infiltrates (CD68+ cells and a smaller CD45Ro+ cell population). Purkinje cell loss and Bergmann gliosis and loss of neurons in the inferior olives
Kinney 1982, USA	1	Brain	Loss of Purkinje cells; atrophy, gliosis of the dentate nucleus, cerebellar granular layer, thalamus, hypothalamus & periaqueductal grey; Senile plaques in the neocortex & hippocampi; Cerebral gliosis subcortical & white matter
Mittelbronn 2010, Germany	1	Brain	Loss of Purkinje cells & cerebellar granular layer cells; Cerebellar atrophy and astrocytic gliosis; Severe neuronal loss inferior olives & accumulation of corpora amylacea. Cerebral reactive astrogliosis and microglial activation Inflammation dominated by CD8+/granzyme B+ & CD20-/CD138- diffuse infiltrates & perivascular cuffing in the cerebellum and brainstem
Nanri 2011, Japan	1	Brain	Loss of Purkinje cells; Minimal cerebellar atrophy; Mild Bergmann gliosis. Empty basket cells, Edematous splitting of Purkinje cell layer, loss of granular cells. No lymphocytic infiltration (CD3-, CD4-, CD8-, CD20-, CD68-, CD79A-)
Tuzun 2001, Turkey	1	Skin	skin biopsy: Periodic Acid Schiff-positive inclusions, diastase resistant intracellular inclusion bodies in apocrine sweat gland cells
Neuropathy 10	37		
Brannagan 2005, USA	8	Skin	Reduced epidermal nerve density, distal > proximal
Cooke 1966, UK (I) *	11	Nerve	Axonal swelling, loss of myelinated fibers, focal proliferation of sarcolemmal nuclei and collateral reinnervation
Cooke 1966, UK (II)			
Chin 2003, USA	3	Sural nerve	Mild to moderately severe chronic axonopathy with loss of myelinated fibers
De Sousa 2006, USA	6	Skin	Morphological changes & a reduced epidermal nerve density
Hadjivassiliou 2006, UK **	3	Sural nerve	Focal inflammatory cell infiltrate in the epineurium & perivascular cuffing of lymphocytes; Patchy loss of myelinated fibers & occasional degeneration
Hadjivassiliou 2010, UK	2	Spinal cord	Degeneration of the dorsal columns; Preservation of the ant.-lat. white matter; Subtotal loss of myelin; Axonal loss; Lymphocytic infiltration
Simonati 1998, Italy	1	Sural nerve	Chronic axonal neuropathy; Significant loss of myelinated fibers & Schwann cell nuclei; Low density of unmyelinated fibers; No inflammatory cells objectified
Souayah 2008, USA	2	Skin	Low to normal epidermal nerve fiber density; Sparse nerve fibers; axonal swelling; increased branching; uneven distribution of epidermal fibers in calf and thigh both
Squintani 2009, Italy	1	Sural nerve	Loss of myelinated axons; Axonal degeneration with focal distribution in different fascicles; Mild perivascular mononuclear cell infiltration of epineural blood vessels; Thickened perineurium
Myopathy 7	36		
Alawneh 2008, Jordan	1	Muscle	Muscle necrosis; Neutrophilic infiltration; Secondary leukocytoclastic vasculitis
Danielsson 2017, Sweden	13	Muscle	Inflammatory infiltrates & muscle fiber degeneration
Hadjivassiliou 1997, UK	2	Muscle	Inflammatory myopathy & Basophilic rimmed vacuoles
Hadjivassiliou 2007, UK	13	Muscle	Internal nuclei; Basophilic rimmed vacuoles; fiber splitting; Endomysial chronic inflammatory cell infiltrate (CD3+ cells), Fibrosis
Hendriksson 1982, Sweden	5	Muscle	Basophilic sarcoplasm; Vesicular nuclei, Muscle Fibre Atrophy, Splitting & Internally placed nuclei
Kleopa 2004, Cyprus	1	Muscle	Inflammatory cell infiltrates; Rimmed vacuoles; Sural nerve biopsy: Chronic active axonopathy; Loss of myelinated fibers; Regeneration & Necrotic fibers
Williams 2003, USA	1	Muscle	Basophilic rimmed vacuoles
Encephalopathy 5	9		
Brucke 1988, Austria	1	Brain	Brain edema; Periventricular lesions; Inflammatory necrosis; Demyelinated fibers; hypertrophy of the inferior olives; Vermal Bergmann gliosis; Lymphocyte infiltration of the pons and mesencephalon
Dimberg 2007, USA	1	Brain	Loss of Purkinje cells & granular layer cells; Astrocytic gliosis of the frontal, parietal, occipital cortices, globus pallidus, hippocampus, midbrain, pons, medulla; Widespread perivascular lymphocytosis in the cortex hippocampus and temporal gyrus, frontoparietal atrophy;
Hu 2006, USA	5	Brain	Non-specific gliosis & astrocytosis; Ubiquitin-positive inclusions in 1 patient
Keller 2006, USA	1	Brain Spinal cord	Loss of Purkinje cells; Neuronal loss of the dentate nucleus; Perivascular cuffing of lymphocytes; Arterial deformations of the pons, midbrain, thalamus and basal ganglia; Mild degeneration of the pyramidal tract & posterior columns; Loss of myelinated nerve fibers in the nerve roots
La Mantia, 1998, Italy	1	Brain	Brain edema; Calcifications, Increased vascularity, Mild neuronal loss, Reactive gliosis & Demyelination
Epilepsy 2	2		
Bye 1993, Australia	1	Brain	Pial angiomatosis consisting of groups of small veins entrapped by collagen; Severe sclerosis of veins; Lymphocytic perivascular cuffing in the cortical neuropil. Atrophy of the white matter and influx of macrophages & reactive gliosis (all occipital lobe)
Orstavik 1997, Norway	1	Brain	Megalencephaly

Post: posterior, lat: lateral, ant: anterior, CNS: central nervous system, * & **: Same article.
